# Motor intervention with and without Nintendo® Wii for children with developmental coordination disorder: protocol for a randomized clinical trial

**DOI:** 10.1186/s13063-019-3930-2

**Published:** 2019-12-30

**Authors:** Jorge Lopes Cavalcante Neto, Bert Steenbergen, Eloisa Tudella

**Affiliations:** 1 Universidade Federal de São Carlos (UFSCar), São Carlos, São Paulo Brazil; 2 Radboud University, Nijmegen, The Netherlands; 3 State University of Bahia (UNEB), Bahia, Brazil

**Keywords:** Developmental coordination disorder, Virtual reality, Nintendo Wii, Motor performance, Motor learning, Motor training, Children

## Abstract

**Background:**

Despite the benefits highlighted by motor interventions based on virtual reality for children with Developmental Coordination Disorder (DCD), there are still doubts as to whether these are greater than those obtained with conventional interventions due to the absence of systematized protocols, and lack of evidence. Here, we present a protocol to systematically compare the effects of two motor-training programs (one Nintendo® Wii-based and the other no-Wii motor activities) on the motor learning in children with DCD.

**Methods/design:**

Two intervention protocols (one based on Nintendo® Wii and the other no-Wii motor activities) will be carried out, with interventions occurring twice a week in 60-min sessions, with a minimum of 12 and a maximum of 16 sessions per child.

The protocols were developed based on the domains of the Movement Assessment Battery for Children – Second Edition (MABC-2) (Manual Dexterity, Aiming and Catching, Balance), with two activities for each of the MABC − two domains. The study will include children aged 7 to 10 years with a total MABC-2 score ≤ 16, and a Developmental Coordination Disorder Questionnaire (DCDQ) score < 46 (age of 7 years), score < 55 (age group of 8 to 9 years and 11 months), or score < 57 (age of 10 years) as scored by the parents. Children will be randomly allocated by draw in one of the two intervention protocols. MABC-2 and DCDQ will be applied before and after intervention to evaluate the effects of the interventions on motor performance and parents’ perception, respectively. Motor learning will be assessed by means of the scores obtained in the games. Evaluators and therapists will be trained and evaluators will be blind regarding the data of the children in the study.

**Discussion:**

Owing to its motivating aspects, training with Nintendo® Wii may be particularly beneficial for children with DCD. The results of this study protocol should help researchers and therapists to better understand the benefits of Nintendo® Wii-based motor intervention over those obtained with no-Wii interventions in children with DCD. It should also create references about more systematized protocols for replication in clinical practice, seeking the improvement of the motor components of these children.

**Trial registration:**

RBR-89ydgj

## Background

Children with compromised motor control are widely mentioned in various studies [1–3], due to the fact that affected motor ability directly or indirectly affects the performance of functional activities. Developmental Coordination Disorder (DCD) has a number of characteristics related to motor development that have a meaningful impact on the daily and school life of many children [4]. DCD diagnostic criteria involve significant motor alterations, compromising daily life, school life or leisure activities, and the fact that these alterations appeared in the earlier stages of children’s life despite sufficient practice of motor activities [5].

Neuroimaging data have highlighted important cortical alterations in children with DCD, specifically in the frontal, parietal, and temporal regions [6, 7] during the performance of manual tasks, which may be associated with a slower processing of motor information in these children when compared to typical ones [8].

Such restrictions may lead to reduced social participation [9] and school performance [10] meaningfully, since many children with DCD tend to isolate themselves from other children because they cannot perform motor activities at the same pace as their peers due to the limitation in information processing. They also tend to become more anxious and insecure [11], and feel more motivated to perform activities when they are alone, as well as those with sedentary characteristics [12].

Motivating strategies in the intervention with these children seem to be the key to success in terms of participation and functional gains [13]. The use of technology has gained prominence in recent years among the strategies adopted since resources, such as interactive games based on virtual reality (VR), offer instant feedback and a larger number of repetitions of body movements per session than many conventional motor intervention techniques, such as physiotherapy, occupational therapy, cognitive orientation to daily occupational performance (CO-OP) or Neuromotor Task Training (NTT) [14]. This facet may promote greater efficacy of the motor intervention in these children.

More specifically, the use of the Nintendo® Wii in the rehabilitation of children with motor alterations has been promoted for ease of measuring the force applied and capturing pressure change available in the Wii Balance Board (WBB), besides the fact that the Wii motion control allows great stimulation of the hand-dexterity motor component [15, 16]. Balance, Aiming and Catching are the domains of the Movement Assessment Battery for Children (MABC), that are regarded as the gold standard for the identification of DCD in children [17]. These aspects make the Nintendo® Wii very useful as a VR resource for motor interventions in children with DCD.

At present, however, little evidence exists in the literature on comparisons between the benefits of VR training, such as Nintendo® Wii, and conventional interventions such as without Wii [18–20].

The handful of existing studies that compare interventions with and without VR for children with DCD still show limitations about evidence created by the VR intervention [19, 21–24]. Moreover, it is not clear whether one type of intervention offers greater gains than the other due to the shortcomings from the scarce previous studies already published. Based on a recent systematic review by Hickman et al. [25] evidence for advantages gained by a VR intervention for children with DCD are not consistent in relation to conventional therapy. The authors argued that due the heterogeneity of assessment tools and outcomes fair comparisons were not possible. So, we believe that in this study we might see fair comparisons because the tasks will be closely matched by motor domain target into the training protocol.

These limitations of present approaches hinder a well-informed conclusion as to whether training with VR is more effective than conventional training in children with DCD. In this paper we present a protocol for a randomized clinical trial to systematically compare two motor-training programs (with and without Nintendo® Wii) on motor learning in children with DCD.

## Methods/design

### Design and general characteristics

**Fig. 1:**
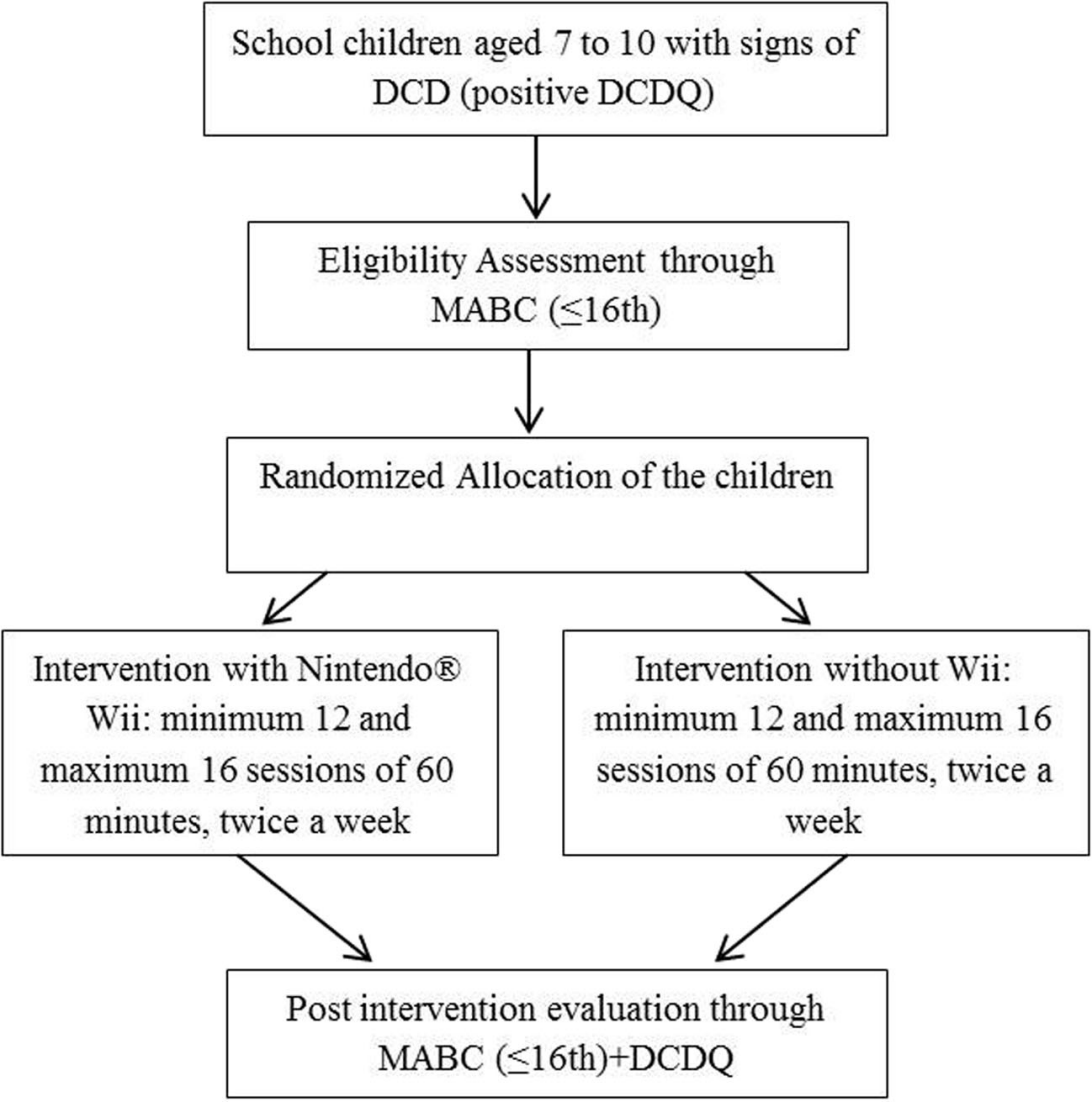
Flow chart of participant inclusion

The protocol consists of a clinical, randomized controlled, blinded trial with two arms: training with the Wii, and conventional training without the Wii. Therapists will undergo instructional training to become familiar with all protocol activities, as well as with the scoring system of each game. The instructional training will occur a month before the beginning of the trial, which has a total duration of 8 h, for 2 h weekly. Despite the rigidly structured task settings required over the sessions, the therapists will be allowed to display their skills as therapist in order to promote a motivational environment for the children during the sessions as well as a great relationship between therapist and patient. There will be different therapists in each of the two intervention protocols, professionals from the areas of physiotherapy, physical education, and occupational therapy. The therapists in the two groups have comparable backgrounds and skill sets. All therapist teams have the same experience with pediatric rehabilitation, around 4 years of experience in this field. Children fulfilling the inclusion criteria in the study will be randomly allocated by draw into one of the two intervention groups. The flow chart of the subjects which will be allocated in the study is shown in Fig. 1 (Additional file 1).

### Patient population

Participants will be children aged from 7 to 10 years who have been diagnosed with Development Coordination Disorder. They will be recruited from public and private elementary schools in the city of São Carlos, State of São Paulo, Brazil. The recruitment process as well as advertising of our study will be based on meetings at schools. If necessary, we will use social media to advertise the project and increase the number of potential children with DCD. Their diagnosis is based on the following *Diagnostic and Statistical Manual of Mental Disorder –V* (*DSM-V*) [5] criteria for DCD:

Criteria A – Children displaying motor performance regarded as low for the age range and for the conditions of practice opportunity. The Movement Assessment Battery for Children – Second Edition (MABC-2) will be used for the evaluation of their motor performance [26]. They will be allocated in the Amber Zone range for DCD with a total score percentage of ≤ 16, or in the Red Zone range with a total score percentage of ≤ 5, to be included in the study.

Criteria B – The limited motor performance of the children interferes significantly with daily life, school life, and leisure activities. The Developmental Coordination Disorder Questionnaire (DCDQ – Brazilian version) [27] will be used to assess, according to the perspective of the parents/caregivers, the loss in children’s functional life, in addition to direct interviews with them about the children’s routine.

Criteria C – Symptoms appeared in the initial developmental stages of the children. Direct interviews with parents/caregivers will be carried out to ascertain such characteristics.

Criteria D – The motor limitations showed by the children do not come from intellectual or visual impairment, or neurologic conditions such as cerebral palsy, dystrophy or any degenerative disease. In order to eliminate these associated conditions, school data about the children will be assessed, teachers will be interviewed and also physiotherapists will offer assessments in order to eliminate any sign of the mentioned neurologic conditions. In addition, psychiatric disorder history will be assessed through the parents’ interview as well as based on children’s forms at school in order to avoid the heterogeneous profile of those children. If we find children who have already received a psychiatric diagnosis (other than DCD) we will exclude them from the study. In addition, we will exclude children who may have undiagnosed psychiatric conditions but have recognizable contra-indications such as high levels of anxiety, impulsivity or attentional difficulties.

Highly anxious, inattentive or oppositional children who might be unable to complete either intervention program will be excluded from the study because they might find the interventions distressing. Children will be allowed to continue regular physical education classes at schools. Other concomitant care or interventions during the period of intervention will not be allowed.

In case any children are missed before the intervention ends, we use the intention-to-treat analysis.

### Interventions

Both intervention protocols are based on the Movement Assessment Battery for Children – Second edition (MABC-2) domains: Manual Dexterity, Aiming and Catching, and Balance. Six games/activities for Nintendo Wii are used that target these domains and six no-Wii activities that are compatible with the activities selected for Wii. The reason for selecting these six of activities is that they address potential improvements in the skills that are required for the standard assessment for children with DCD. The second reason was to render the games/activities as close as possible to those required for MABC-2 evaluation in terms of movement standard.

Each game/activity will continue for 7 min and in total 42 min will be spent on the six activities. With the purpose of exchanging materials and equipment from one game to the next, thus allowing to complete the total time of 60 min per session.

Sessions of both protocols will be performed twice a week, with duration of 60 min per session, and with a minimum of 12, and a maximum of 16 training sessions in total. The first session will familiarize the children with the games/activities. The second sessions consists of a pre-test and the last the post-test for motor learning outcome. The number of mistakes, hits, and respective score will be counted in each one of the games/activities. Pre-post scores for each training condition will be compared. The two protocols each have six activities. For the protocol with Wii, existing games will be use and for the no-Wii protocol ‘real life’ activities will be used that target the same domains as the Wii tasks.

The total score for each session will be the sum of the points obtained in all the attempts performed. The sequence of the games will be randomized, with a selection among 16 possible combinations, considering two activities for the same domain in sequence in all of these combinations (Tables 1, 2 and 3).

**Table 1:** Intervention protocol sequence of activities

Session number	Activity 1	Activity 2	Activity 3	Activity 4	Activity 5	Activity 6
1	Bow and arrow/archery	Bowling	Frisbee	Table tennis	Balance disk/marble balance	Balance beams/tightrope walk
2	Bow and arrow/archery	Bowling	Balance disk/marble balance	Balance beams/tightrope walk	Frisbee	Table tennis
3	Table tennis	Frisbee	Bowling	Bow and arrow/archery	Balance disk/marble balance	Balance beams/tightrope walk
4	Balance beams/tightrope walk	Balance disk/marble balance	Table tennis	Frisbee	Bow and arrow/archery	Bowling
5	Balance disk/marble balance	Balance beams/tightrope walk	Frisbee	Table tennis	Bowling	Bow and arrow/archery
6	Table tennis	Frisbee	Bow and arrow/archery	Bowling	Balance beams/tightrope walk	Balance disk/marble balance
7	Bow and arrow/archery	Bowling	Balance beams/tightrope walk	Balance disk/marble balance	Table tennis	Frisbee
8	Bow and arrow/archery	Bowling	Table tennis	Frisbee	Balance disk/marble balance	Balance beams/tightrope walk
9	Balance beams/tightrope walk	Balance disk/marble balance	Frisbee	Table tennis	Bowling	Bow and arrow/archery
10	Frisbee	Table tennis	Bow and arrow/archery	Bowling	Balance disk/marble balance	Balance beams/tightrope walk
11	Balance beams/tightrope walk	Balance disk/marble balance	Bow and arrow/archery	Bowling	Table tennis	Frisbee
12	Bowling	Bow and arrow/archery	Frisbee	Table tennis	Balance disk/marble balance	Balance beams/tightrope walk
13	Balance beams/tightrope walk	Balance disk/marble balance	Bowling	Bow and arrow/archery	Frisbee	Table tennis
14	Balance disk/marble balance	Balance beams/tightrope walk	Bowling	Bow and arrow/archery	Frisbee	Table tennis
15	Bowling	Bow and arrow/archery	Balance disk/marble balance	Balance beams/tightrope walk	Frisbee	Table tennis
16	Frisbee	Table tennis	Bowling	Bow and arrow/archery	Balance disk/marble balance	Balance beams/tightrope walk

**Table 2:** Training activities with Wii and the three domains

Manual Dexterity	Aiming and Catching	Balance
Frisbee	Bowling	Tightrope walk
Table tennis	Archery	Marble balance

**Table 3:** No-Wii Experimental protocol activities

Manual Dexterity	Aiming and Catching	Balance
Frisbee	Bowling	Balance disk
Table tennis	Bow and arrow	Balance beams

### Experimental protocol with Wii

This protocol was based on percepto-motor activities, consisting of activities and games based on VR, supported by Nintendo® Wii resources: the Wiimote control and the WBB platform, communicating via bluetooth. The training activities are detailed below:

### Frisbee

This activity will be performed with the children holding the Wiimote control in the fist, in a space of 1.10 m^2^, at a 2-m distance from the television screen to which the Nintendo® Wii will be connected, performing throws of the virtual Frisbee, aiming to hit the target in the scoring area projected.

Children will be encouraged to perform the highest number possible of throws in the period of 7 min, trying to hit the scoring areas worth 10, 50, and 100 points. Every time the Frisbee hits these spaces, it will count as a hit and as a mistake each time it hits outside them. The sum of all attempts hitting any scoring area during 7 min in each session will be the basis of the score.

### Table tennis

This activity will be performed with the children holding the Wiimote remote control in their fist, and they will be encouraged to use it as if it were a table tennis paddle, to serve, hit and hit the ball again, trying to score more points than the opponent (virtual opponent chosen by the machine). Each game will automatically end when one of the players scores 8 points. Each point scored by the children will count as a hit and those scored by the opponent (machine) as a mistake. The children must score the maximum number of points in 7 min.

### Bowling

This activity will be performed with the Wiimote control held in the fist, which serves as if it were the bowling ball. The aim is to perform the throws and try to knock down the highest number of pins. Two blocks of nine attempts will be performed. At the end of the 10th block, children will start a new session of two attempts each until the end of the 7-min period. Each throw knocking pins down will count as a hit, or as a mistake in the opposite case. Children who knock down all 10 pins at once will be considered Strikers and will double the score for the next throw, while those knocking down the 10 pins in two throws (first to ninth blocks) or three (10th block) will be considered a Semi-Striker, receiving half of the score they achieve in the next throw as a surplus.

### Archery

This activity will be performed with the Wiimote remote control held in the children’s fist. The movement to be performed with the Wiimote should represent the action of a bow moving the arrow to reach the aim with different scoring areas, varying from 1 to 10 points. The children, in the figure of an avatar, will go through three stages of the game at the beginner level, each one with different scenery and distances (10 m, 25 m, and 35 m) between avatar and aim.

At the end of the three stages of attempts, children will return to the beginning stage and continue to perform new attempts until the end of the 7-min period. Each arrow shot and sticking to the target will count as a hit, and as a mistake in the opposite case. The total score for each session will be the sum of the points obtained in all the attempts performed.

### Tightrope walk

This activity will be performed with the WBB equipment, fixed to the ground, in a space of 1.10 m^2^ and at a distance of 2 m from the television screen. Children will have to step with both feet on the equipment and perform the action of the game, consisting in a 35-m tightrope walk between the top of virtual buildings. As a form of equalizing scores, the total length of the rope is divided in three parts: the two first of 12 m each and the last of 11.

The basic movement that children will perform is raising their feet alternately, moving the trunk sideward so that the avatar maintains the balance as it walks on the rope. Each third part of the rope walked without falling will count as a hit, while any fall in lengths shorter than 12 or 11 m will count as a mistake. The first 12 m walked will be worth 15 points, the 12 intermediary meters will be worth 20, and the last 11 m 10 points.

### Marble balance

This activity will be performed with the WBB equipment fixed to the ground, similar to what described for the tightrope walk. Children will move their trunk to different positions while trying to throw the ball in the holes of the virtual platform, which will be turning all the time, provoking greater unbalance and difficulty in the game. Every ball falling out of the virtual platform will count as an error, and each one in the respective hole will be worth a point. As the balls disappear, the children will move to a new platform, with more balls to be thrown into the holes. Scores will be calculated by the Nintendo® Wii equipment itself with basis on the performance of the avatar commanded by the children.

### Experimental protocol no-Wii training

This protocol was based on a proposal of percepto-motor type, and named no-Wii training. Its training protocol is detailed on the following table:

### Frisbee

**Fig. 2:**
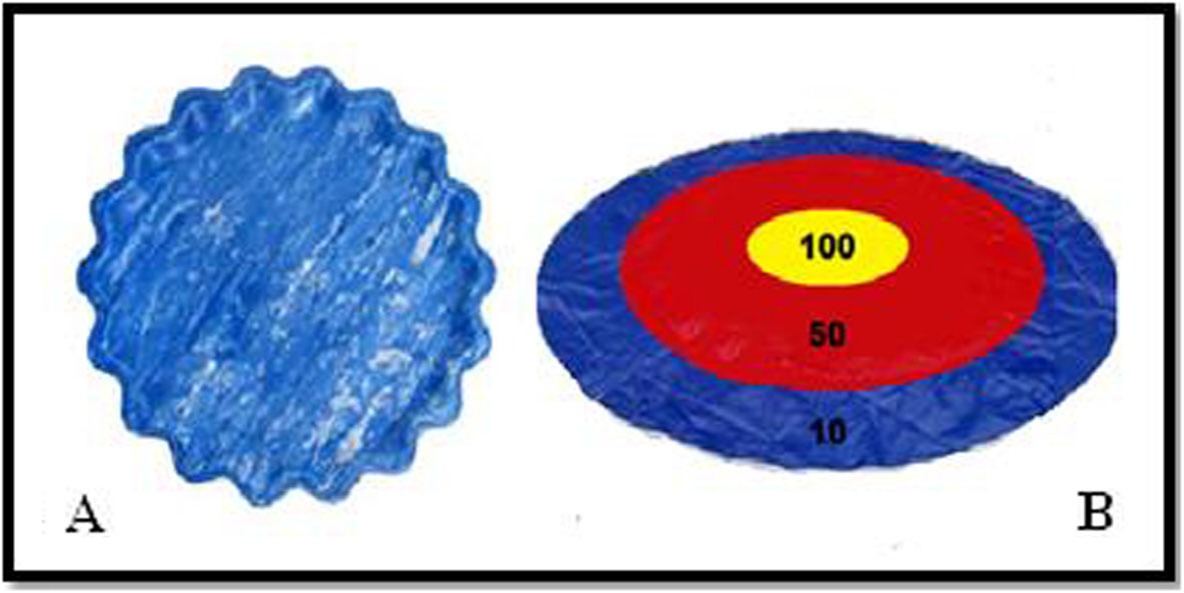
**a** Frisbee, **b** Frisbee target

The game of Frisbee consists in throwing a Styrofoam disk measuring 24 cm of circumference (Fig. 2a) on a paper card target with a circumference of 2.82 m and placed at a distance of 4 m (Fig. 2b). The target must be fixed to the ground and display three different scoring areas (10, 50, and 100 points).

### Table tennis

**Fig. 3:**
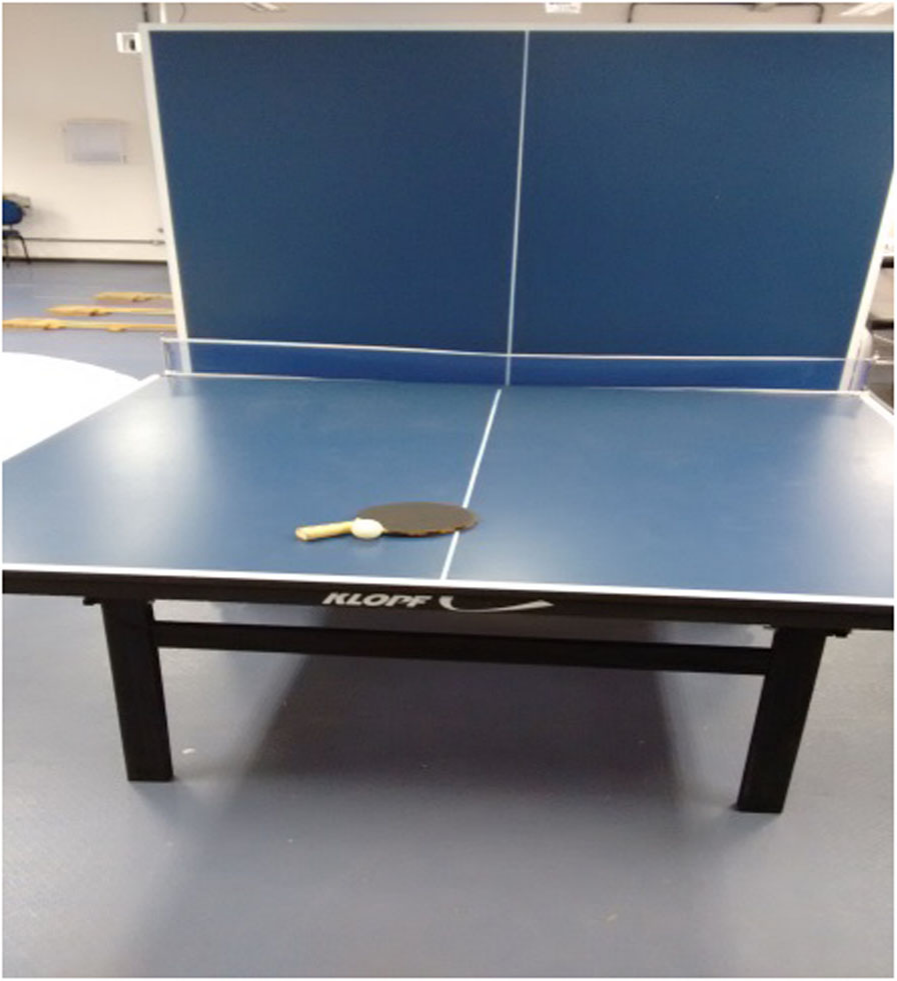
Tennis table

The game of table tennis will be performed with the elevation of one of the two halves of the table, allowing children to throw and hit the ball again with their paddle (Fig. 3). The aim of this activity consists in making the ball hit the raised half of the table after it hits the horizontal part and going back to the same side, and children will be given a hit for each movement of this kind. Balls falling out of the game area, multiple rebouncing on the horizontal part of the table and a serving or reception error will count as mistakes.

### Bowling

**Fig. 4:**
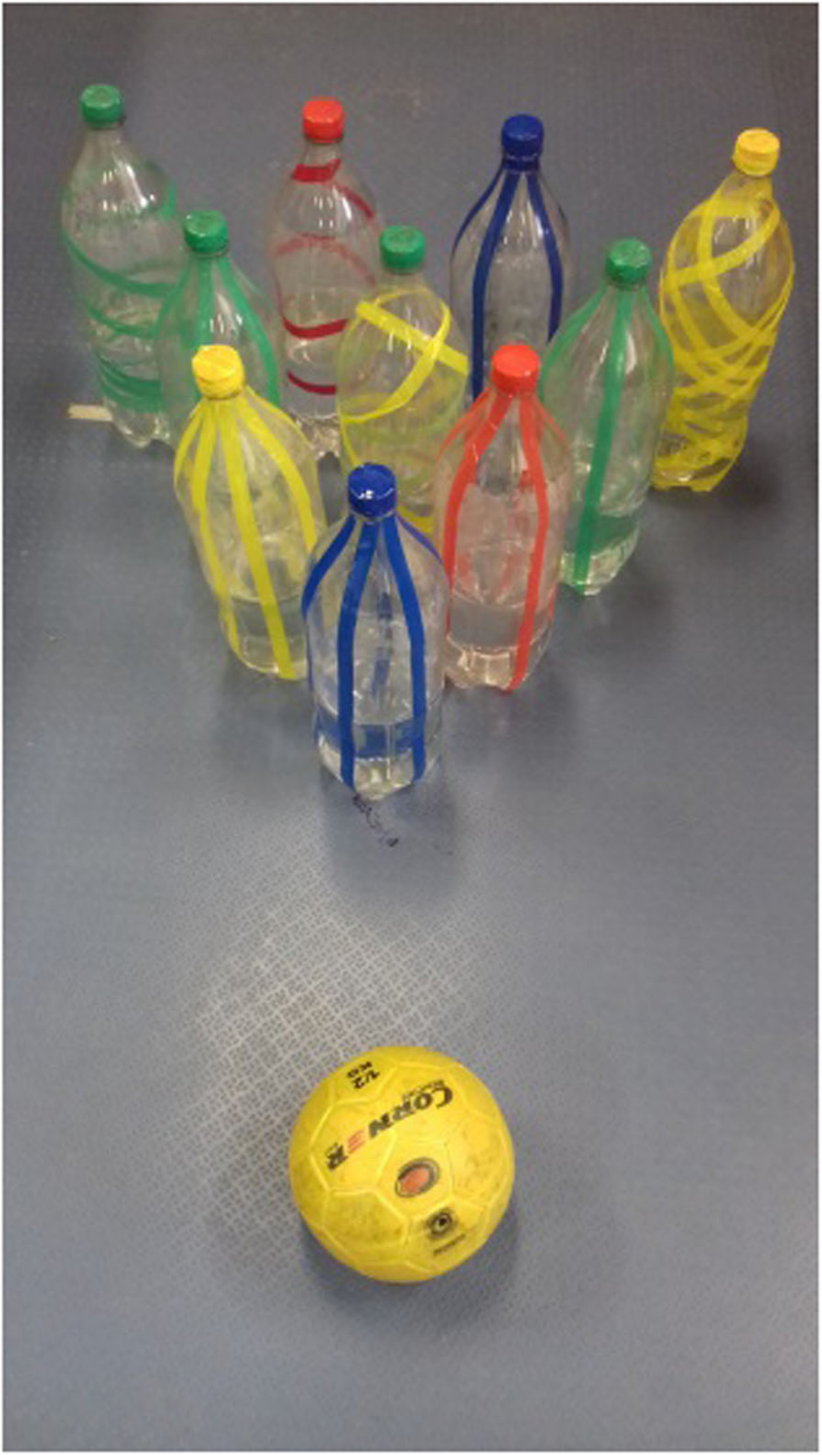
PET bottles and ball in the bowling target area

The game of bowling will be performed with the use of 10 PET bottles containing 600 ml of water each (Fig. 4), placed at a distance of 4.83 m for children aged 7 and 8 years, and at a distance of 6.03 m for those aged 9 and 10 years. The aim of the activity is to throw a 0.5-kg medicine ball towards the bottles and knock down the highest number possible at once (Fig. 4).

Similar to conventional bowling, children will be conceded two throws per block of attempts, considering that in the 10th block the children will have the right to three attempts. The bottles knocked down in the first attempt of each block will be put in place again only after the children have concluded the throws in the second or third attempt (only for the 10th block). Each bottle knocked down will be worth a point. Throws failing to knock down pins will count as zero in the score and as mistakes, while successful attempts will be regarded as hits.

If children manage to knock down all bottles at once, they will achieve the Striker score, doubling it in the following attempt, and if they knock down all the bottles in two attempts of a block or in the three attempts of the 10th block they will attain the Semi-Striker score, receiving half the score of the following throw as surplus.

### Bow and arrow

**Fig. 5:**
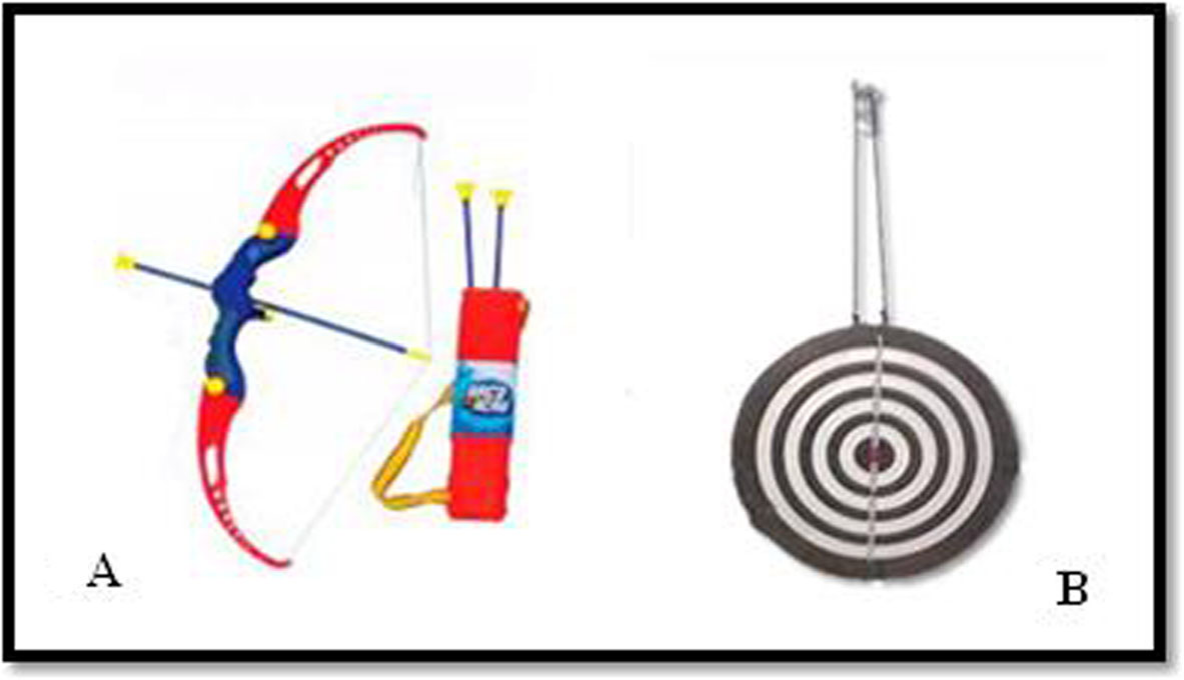
**a** Bow and arrow, **b** Target for bow and arrow

This activity will be performed with a plastic bow-and-arrow commercial set (Fig. 5a), including a 62-cm bow and the arrows, each one of 41 cm. The arrows end with a suction cup which sticks to the target (Fig. 5b), to indicate the score that each child achieves in the game.

A wooden target, covered with an adherent plastic surface so that the suction cups at the tip of the arrows stick to it, will be used. It measures 41 cm in circumference, and it will be fixed to the wall with a 120-cm adjustable rope in front of the children, at their visual height, at a distance of 2.30 m for 7- and 8-year-old children and 2.97 for children aged 9 and 10 years. Each arrow hitting the target and sticking to a scoring area will count as a hit, while those not fixing to it as a mistake. Scores will be based on the scale displayed on the target itself, ranging from 1 to 10 points.

### Balance disk

**Fig. 6:**
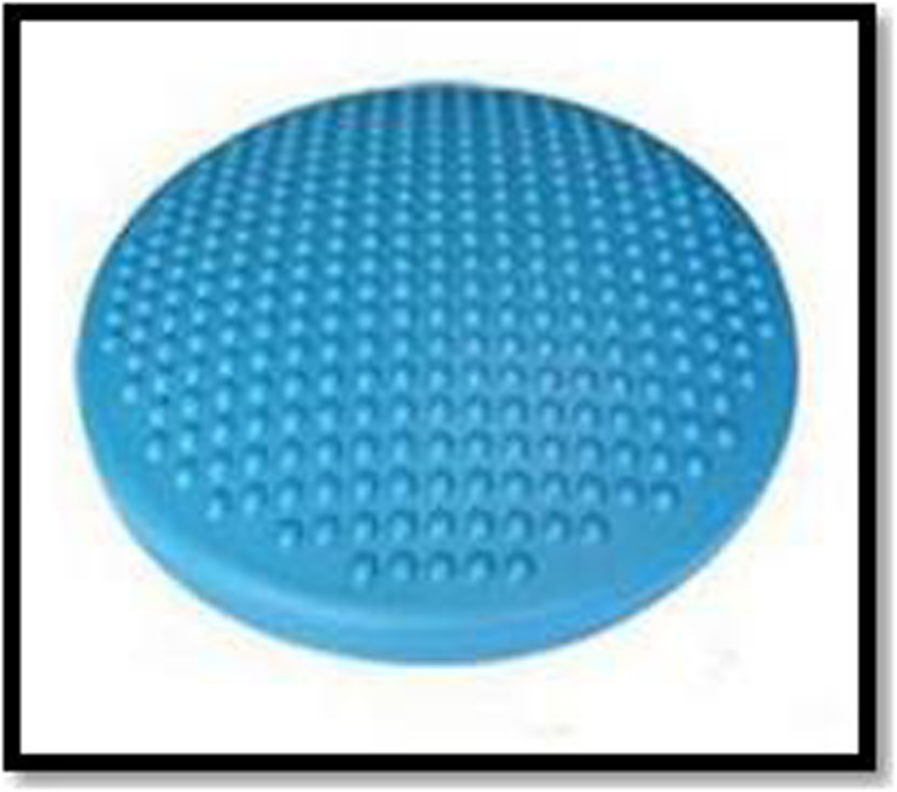
Balance disk

This activity will be performed with a vinyl balance disk measuring 40 cm in circumference (Fig. 6) placed on the ground; the children will perform 14 different static balance postures on top of it (Table 4). The postures were based on, and adapted from, the Berg Balance Scale [28]. Children aged 7 and 8 years must maintain the postures for 15 s, while 9- and 10-year-old children must keep them for 30 s. Each posture performed correctly and for the time due will count as a hit, while unbalances, falls, stepping on feet or putting hands on the ground will be regarded as mistakes. In the second case, children must go to the following posture. Each hit will be worth 10 points, and at the end of the 14 postures, they will repeat the same sequence until the time is over.

**Table 4:** Sequence of movement for balance disk

Sequence of movements – Balance disk	
1 – Standing, without support, with feet shoulder-width apart	
2 – Standing, without support, feet together	
3 – Standing, without support, with preferential foot on the disk and the other suspended in the air (90° knee flexion)	
4 – Standing, without support, with unpreferential foot on the disk and the other suspended in the air (90° knee flexion)	
5 – Standing, without support, with preferential foot forward	
6 – Standing, without support, with unpreferential foot forward	
7 – Standing, without support, with preferential leg raised forward	
8 – Standing, without support, with unpreferential leg raised forward	
9 – Standing, without support, with preferential leg in hip abduction	
10 –Standing, without support, with unpreferential leg in hip abduction	
11 – Standing, without support, with feet shoulder-width apart, trunk flexion and arms forward	
12 – Standing, without support, feet together, trunk flexion and arms forward	
13 – Standing, without support, with feet shoulder-width apart and eyes closed	
14 – Standing, without support, feet together and eyes closed	

### Balance beams

**Fig. 7:**
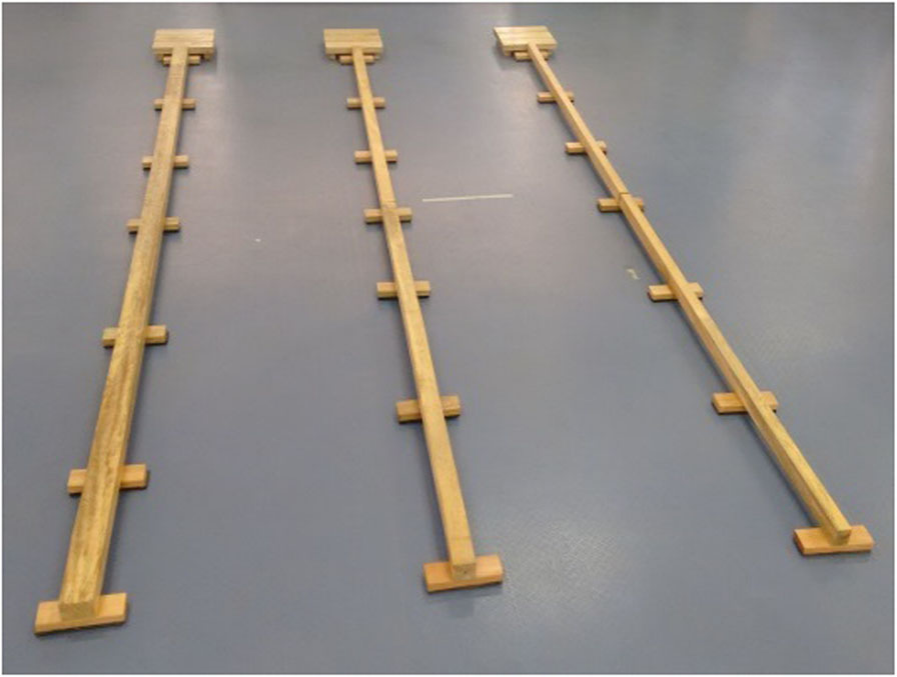
Balance beams

This activity will be performed with three 3 m-long wooden beams, measuring 3 cm in height, parallel to the ground (Fig. 7), each one with a different width, therefore offering children various degrees of difficult. The widest stick measures 6 cm, the intermediary 4.5 cm and the narrowest 3 cm. Three wooden traverses measuring 15 × 1.5 × 5 cm will be placed at the beginning of each beam, 50 cm apart from one another. Children will have to move on each one of the beams, from the widest to the narrowest, stepping with one foot at a time, so that the heel of the front foot is close to the toes of the back foot.

Each beam stepped correctly will count as a hit, and any fall, using the beam or the ground as a support, slipping or losing contact of the feet with the beam, touching the ground or not performing the steps with feet close will count as an error. Scores for beams stepped correctly are as follows: 10 points (6-cm wide beam), 15 points (4.5-cm wide beam) and 20 points (3-cm wide beam). At every unsuccessful attempt, children will go back to the beginning of the beam where the mistake occurred and start the route again, moving to the next beam after doing the correct movement on the previous one.

### Outcomes measures

All tests will be applied by trained evaluators, blind regarding the allocation of the children in the intervention groups. Evaluators will be professionals in the areas of physiotherapy, physical education, and occupational therapy. Each child will be assessed by the same person before and after treatment.

### Primary outcome measure

#### The primary outcomes of this study is the motor performance

##### Motor performance – MABC-2

The motor performance of the children will be assessed through the MABC-2 total standard score (TSS). As mentioned before, MABC-2 is considered a gold standard tool to identify motor performance problems in children from 3 years old. MABC-2 is divided in three different age bands: the band 1 for children aged from 3 to 6 years old; band 2 for children aged from 7 to 10 years old; and band 3 for children aged from 11 to 16 years old. There are eight tasks in total, three for the Manual Dexterity domain, two for the Aiming and Catching domain; and three for the Balance domain. Children received a specific score in each domain and their motor performance classification is based on total score and percentiles values. Children < 5° percentile present motor difficult; children < 16° percentile are at risk for motor difficult and children > 16° percentile have no motor difficult detected. According to the MABC-2 tester manual, each child spends about 30–40 min completing all tasks.

### Secondary outcomes measures

#### Motor learning

Motor learning in children will be assessed by means of the score obtained in the games. Individual scores in each of the six games from the second session (pre-test) will be summated as well as those from the six games of the last session (post-test) to obtain the general measurement of motor learning due to the treatment.

Individual scores in each game and for domain will also be counted (Manual Dexterity, Aiming and Catching, and Balance), before and after intervention. With the purpose of analyzing whether some activities are better learned in one type of intervention than in another. A specific form will be available for each therapist fills this information in each session.

### Signs indicating DCD – Developmental Coordination Disorder Questionnaire (DCDQ)

The DCDQ [27] will be used with parents/caregivers of the children, who will fill in the questionnaire before they begin the treatment and also after the end, seeking to verify possible changes in parents’ perception about the motor characteristics of the children. We expect that the parents will fill the DCDQ in about 10–15 min.

### Anthropometric measurements

On the same day of the MABC-2 pre-test evaluation, anthropometric data about BMI (Body Mass Index), body fat percentage (BF%) and waist circumference (WC) of the children will be collected. Weight measurements for BMI will be made with a portable digital W602 (WISO®) scale, and height will be measured with a portable Wood (WCS®) stadiometer.

BMI will be calculated through the height/weight relation [29]. To calculate the BF% of the children, measurements around triceps and the subscapular skinfold will be done with a Lange® adipometer. Waist circumference will be measured in the narrowest region between the last rib and the umbilicus with a Sanny® metric tape. All anthropometric measurement collection procedures will be done in the observance of the techniques standardized by the literature. The anthropometric measurements will be performed in about 10–15 min.

### Motivation – Enjoyment Scale

At the end of the last session, the perception of satisfaction with each one of the activities and with the intervention as a whole will be assessed for each child through the Enjoyment Scale, an adapted scale by Jeslma et al. [20] translated to Portuguese. The score is expressed by the number of smileys, ranging from 0 (no fun at all) to 4 (super fun). Using this scale, each therapist will have direct results about the impact that the activities and the treatment as a whole had on each child. The Enjoyment Scale will be performed in about 5 min.

### Ethical approval

The respective research project of the present intervention protocols was approved by the Human Research Ethics Committee of the Universidade Federal de São Carlos (CEP/UFSCar), São Carlos, São Paulo, Brazil, with CAEE approval number 47091115.0.0000.5504. This project will be carried out according to the 466/12 Resolution of Conselho Nacional da Saúde (National Health Council), Brazil, and will follow the ethical principles of the Helsinki Declaration. All parents/caregivers will sign the Free and Clarified Consent Term, and the children the Consent Term, being aware of the research aims and procedures and the voluntary participation in the research. According to the Brazilian Ethics Statements, parents and children can stop the training anytime, without any punishments. This clinical trial is registered in the Registro Brasileiro de Ensaios Clínicos (REBEC), www.ensaiosclinicos.gov.br (RBR-89YDGJ); registration date 5 January, 2016, 11:42 p.m. (Brazilian time), last update 21 October 2016, 4:53 p.m. (Brazilian time), version number 1.

### Sample size

Sample size was calculated using the G*Power Software (3.1.9.2 version, Germany) based on a pilot study (*n* = 6). The calculation was performed considering the within-group comparisons for the Wii group, a power of 80%, and an alpha of 5%. The MABC total score assessed before and after 12 sessions was considered the main outcome and then considered for the analysis. The effect size was 0.80 (baseline: 58.0 ± 8.7; post 12-session intervention: 65.5 ± 9.9). Therefore, a sample of 15 participants was required for each group. In order to account for possible dropouts, we increased the sample to 16.

### Randomization

Children will be randomly distributed by draw into one of the two training groups within a week at most after MABC-2 assessment. In case the draw leads to a sequence of four children in the same treatment group, the following four will be automatically allocated in the other. Once the distribution of blocks of four children is equalized, a new draw will be done for the following children to be randomly distributed. A person not involved with the study will receive a list of all children and will write in one piece of paper the term “Wii” and in another piece of paper the term “no-Wii.” This person will fold each piece of paper and will request to another collaborator not involved in this study to choose between the two papers. Then, following this draw, each child will be allocated into Wii or the no-Wii intervention group.

### Blinding

Assessors will be blinded about children’s allocation group. In this sense, the draw for children randomization will be carried out by a professional not involved in the assessments and interventions of the project. Blind statistical analyses will be performed, having children’s names and intervention groups modified by numeric codes in the spreadsheets.

### Statistical methods

At first, descriptive analysis will be done with the distribution of relative and absolute frequencies, averages, standard deviations, medians, mode, and minimum and maximum number in order to characterize children. All data will be inserted into spreadsheets in the SPSS statistical package, version 20.0 for Windows.

Next, analyses regarding normality and homogeneity tests will be performed. There will be two independent groups because of the design of the study, and there will also be intergroup comparative analyses in each one, constituting the results of motor performance and learning evaluation pre test and post test, as well as the comparisons between such intergroup evaluations.

In case of parametric distribution, analyses will be performed through analysis of variance (ANOVA) and analysis of co-variance (ANCOVA) utilizing the Bonferroni correction.

In case of non-parametric distribution, intergroup comparative analyses will be carried out by means of the Mann-Whitney *U* test and intergroup comparison through the Wilcoxon test. The significance level adopted will be *p* < 0.05 for all measurements. In order to verify the dimension of the effect of the interventions realized, the Cohen test will be applied with the following reference values: *d* = 0.3 indicates a small dimension of the effect, while *d* = 0.5 and *d* = 0.8 indicate a moderate and a large one [30], respectively.

### Study organization

**Fig. 8:**
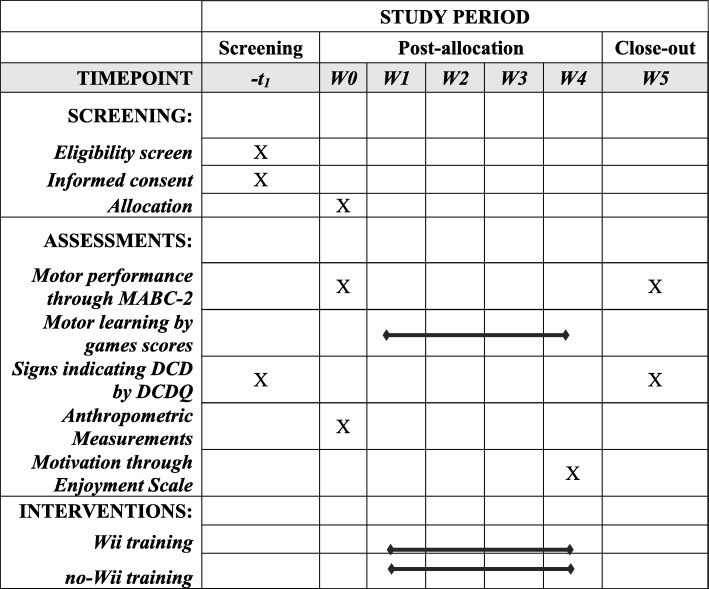
Description of screening, assessment, and intervention phases related to the study

The present study will be performed at the Physiotherapy Department of the Universidade Federal de São Carlos (DFisio/UFSCar), in the city of São Carlos, São Paulo, Brazil. Municipal and State Education secretaries of São Carlos are partners in this project, since they authorized the access of researchers to the schools to recruit the children target of this study, which counts on the financial support of the Fundação de Amparo à Pesquisa do Estado de São Paulo (FAPESP), with process number 2015/24291–0. There is no other role of this funder in the design of this study. A data and safety monitoring committee is not required in this study. The main information regarding screening, assessments, and interventions is available in Fig. 8.

## Discussion

This study protocol is the first to directly compare the efficacy of two motor-training protocols in children with DCD. The protocol is based on the domains of the MABC-2 domains. Various studies have already demonstrated the efficacy of intervention programs based on VR for these children [19–24, 31–36], but the absence of further protocol systematization has been an important hindrance for professionals and researchers in this field to clearly understand the impact of such interventions on children’s activity performance.

Studies comparing interventions based on VR and conventional interventions [19, 22–24] are scarce, and their results tend to demonstrate that despite the benefits of VR, conventional interventions seem to be more effective in the improvement of motor performance of children with DCD [19, 22–24]. Due to limitations and differences between the protocols adopted, though, these results must be interpreted carefully. Hence, when testing the efficacy of these two type of intervention programs, planned in similar manner, with similar scoring systems and activities, it will be possible to enhance evidence about the benefits in motor performance and learning of one program over the other.

One documented advantage of VR training, such as the Wii intervention, is the motivating aspects because the projected architecture on the environment of VR is capable of hold the children’s attention, being an important aspect for rehabilitation of children with DCD [20].

## Additional file

**Additional file 1.** Standard Protocol Items: Recommendations for Interventional Trials (SPIRIT) 2013 Checklist: recommended items to address in a clinical trial protocol and related documents.

## Data Availability

The data that will be collected in this study will be available from the principal researcher.

## Abbreviations

ANCOVA: Analysis of co-variance
ANOVA: Analysis of variance
BF%: Body fat percentage
BMI: Body Mass Index
CO-OP: Daily occupational performance
DCD: Developmental Coordination Disorder
DCDQ: Developmental Coordination Disorder Questionnaire
DFisio/UFSCar: Physiotherapy Department of the Universidade Federal de São Carlos
*DSM-V*: *Diagnostic and Statistical Manual of Mental Disorder –V*
FAPESP: Fundação de Amparo à Pesquisa do Estado de São Paulo
MABC: Movement Assessment Battery for Children
MABC-2: The Movement Assessment Battery for Children – Second Edition
NTT: Neuromotor Task Training
REBEC: Registro Brasileiro de Ensaios Clínicos
VR: Virtual reality
WBB: Wii Balance Board
WC: Waist circumference
